# Clinical Manifestations of Emerging *Trichosporon* spp. Infections, France

**DOI:** 10.3201/eid3201.250504

**Published:** 2026-01

**Authors:** Marie Desnos-Ollivier, Alexandre Alanio, Maud Gits-Muselli, Karine Boukris-Sitbon, Agathe Bertho, Aude Sturny-Leclère, Emilie Guemas, Philippe Poirier, Christine Bonnal, Marie-Elisabeth Bougnoux, Sophie Brun, Taieb Chouaki, Nicole Desbois-Nogard, Elisabeth Chachaty, Florence Persat, Marc Pihet, André Paugam, Françoise Botterel, Magalie Demar, Loïc Favennec, Florent Morio, Frédéric Gabriel, Arnaud Fekkar, Jean-Pierre Gangneux, Caroline Mahinc, Valérie Letscher-Bru, Laurence Millon, Frédéric Dalle, Julie Bonhomme, Muriel Nicolas, Boualem Sendid, Milène Sasso, Laure Courtellemont, Anne-Laure Roux, Estelle Perraud-Cateau, Juliette Guitard, Edith Mazars, Olivier Lortholary, Fanny Lanternier

**Affiliations:** Institut Pasteur, Paris, France (M. Desnos-Ollivier, A. Alanio, K. Boukris-Sitbon, A. Bertho, A. Sturny-Leclère, O. Lortholary, F. Lanternier); Hôpital Saint-Louis, Paris (A. Alanio); Hôpital Robert Debré, APHP, Université Paris Cité, Paris (M. Gits-Muselli); Université Paris Cité, INSERM, IAME, Paris (M. Gits-Muselli); Centre National de la Recherche Scientifique, Toulouse, France (E. Guemas); Centre Hospitalier Universitaire Clermont-Ferrand, Clermont-Ferrand, France (P. Poirier); Université Blaise Pascal, Clermont-Ferrand (P. Poirier); Hôpital Bichat–Claude-Bernard, Paris (C. Bonnal); Hôpital Necker-Enfants Malades, Paris (M.-E. Bougnoux, O. Lortholary, F. Lanternier); Hôpital Avicenne, Bobigny, France (S. Brun); Centre Hospitalier Universitaire Amiens-Picardie, Amiens, France (T. Chouaki); University Hospital of Martinique, Fort-de-France, Martinique (N. Desbois-Nogard); Gustave Roussy, Villejuif, France (E. Chachaty); Centre Hospitalier Universitaire de Lyon, Lyon, France (F. Persat); CHU d'Angers, University Angers, University Brest, GEIHP, SFR ICAT, Angers, France (M. Pihet); Cochin Port Royal University Hospital AP-AP, Paris (A. Paugam); Université Paris-Est Créteil, Créteil, France (F. Botterel); Centre Hospitalier Andree Rosemon, Cayenne, Guyana (M. Demar); University Hospital Charles Nicolle, Rouen, France (L. Favennec); Nantes University Hospital, Nantes, France (F. Morio); Centre Hospitalier Universitaire de Bordeaux, Bordeaux, France (F. Gabriel); Centre d’Immunologie et des Maladies Infectieuses, Paris (A. Fekkar); Hôpital Pontchaillou, Rennes, France (J.-P. Gangneux); Centre Hospitalier Universitaire de Saint-Etienne, Saint-Etienne, France (C. Mahinc); Hôpitaux Universitaires de Strasbourg, Strasbourg, France (V. Letscher-Bru); University Hospital J Minjoz, Besancon, France (L. Millon); Dijon University Hospital, Dijon, France (F. Dalle); Centre Hospitalier Universitaire Côte de Nacre, Caen, France (J. Bonhomme); Centre Hospitalier Universitaire de Guadeloupe, Pointe-à-Pitre, Guadeloupe, France (M. Nicolas); Lille University Hospital Center, Lille, France (B. Sendid); Centre Hospitalier Universitaire Nîmes et Université de Montpellier, Nîmes, France (M. Sasso); Centre Hospitalier Universitaire d’Orléans, Orléans, France (L. Courtellemont); Assistance Publique-Hôpitaux de Paris, Boulogne-Billancourt, France (A.-L. Roux); Centre Hospitalier Universitaire Poitiers, Poitiers, France (E. Perraud-Cateau); Hôpital Saint-Antoine, Paris (J. Guitard); Centre Hospitalier de Valenciennes, Valenciennes, France (E. Mazars)

## Abstract

Fungi in the family Trichosporonaceae are rarely involved in invasive disease but are frequently associated with colonization or respiratory allergic infection. Trichosporonaceae exhibit intrinsic resistance to echinocandin antimicrobial drugs, posing challenges for treatment and contributing to high mortality rates. We complied a nationwide analysis of 112 cases of invasive disease caused by *Trichosporon* spp. and related fungi, diagnosed in France over 20 years, that combined clinical data, susceptibility profiles, and molecular identification. We identified 12 species; *T. asahii* was the most common species recovered, and the new species *T. austroamericanum* was next. Comparison of clinical data highlighted species and genotypic differences, such as a much higher proportion of children infected by *T. asahii* and major differences in antimicrobial drug susceptibility. Correct identification is not only of epidemiologic interest but also necessary for patient management because of the varying clinical and microbiological characteristics found in different species.

Among the wide range of fungi responsible for invasive infections in humans, species within the Trichosporonaceae family are generally considered rare or uncommon pathogens (*1*–*4*). However, recent reports indicate an increasing incidence in both high-income and resource-limited countries over the past decade (*2*,*4*–*6*).

Trichosporonaceae is a family of basidiomycetous, yeast-like fungi that produce arthroconidia and are commonly found in air, soil, and water worldwide (*7*). Trichosporonaceae can also be recovered from contaminated materials or medical devices. Some species are part of the normal or transient human microbiota, particularly on the skin and in the gastrointestinal tract (*8*–*11*). After taxonomic revisions, clinically relevant Trichosporonaceae are now distributed into 3 genera, *Trichosporon*, *Apiotrichum*, and *Cutaneotrichosporon*. Each genus contains multiple species known to cause invasive infections in humans (*12*–*14*). *T*. *asahii* remains the most frequently reported species responsible for invasive fungal disease (IFD) caused by Trichosporonaceae (*5*,*6*,*9*,*15*–*17*), but it is also known to cause summer-type hypersensitivity pneumonitis, particularly in Japan (*18*,*19*). This species is also considered the most virulent among Trichosporonaceae and demonstrates higher MICs for amphotericin B and azoles than other fungi (*15*,*17*,*20*–*22*). The distribution of other species varies, but typically *Cutaneotrichosporon dermatis*, *T. asteroides*, *C. mucoides*, *T. inkin*, and *T. faecale* are relatively commonly involved in IFD (*2*,*4*,*5*,*17*,*21*,*23*,*24*), and *T. inkin* is recognized as a leading cause of white piedra (*25*).

IFD caused by Trichosporonaceae has a high mortality rate, ranging from 30% to 90% (*4*,*24*,*26*,*27*). Such infections are commonly associated with hematologic malignancies (*1*,*2*,*28*) and are frequently reported in pediatric populations (*26*). Immunosuppressed patients are particularly at risk, especially those with central venous catheters, urinary or peritoneal catheters, broad-spectrum antimicrobial drug use, corticosteroid therapy, intensive care unit (ICU) stays, or prior exposure to echinocandins such as caspofungin, to which Trichosporonaceae fungi are intrinsically resistant (*1*,*3*,*4*,*23*,*29*,*30*). Clusters of infection have been described previously (*16*). Current treatment guidelines recommend voriconazole monotherapy for IFD caused by Trichosporonaceae (*28*,*31*), although other azoles and amphotericin B are also commonly used. Some studies advocate combination therapy with amphotericin B and voriconazole (*17*,*20*).

Despite the clinical relevance of Trichosporonaceae infections, epidemiologic data from multicentric studies remain scarce (*4*), and most information stems from case reports or small patient cohorts (*6*,*27*). Because of the variability in antimicrobial drug susceptibility across species, accurate identification is essential for both epidemiologic understanding and clinical management (*1*,*21*). In this article, we report 112 episodes of IFD caused by Trichosporonaceae, diagnosed during 2002–2022 in France. The cases were documented across 41 centers and are supported by clinical data, precise molecular identification, and antimicrobial susceptibility testing.

## Materials and Methods

### Clinical Data

We included in the study all first episodes of IFD caused by Trichosporonaceae notified at the National Reference Center for Invasive Mycoses and Antifungals (NRCMA) during 2002–2022. Demographic and clinical data concerning the patients were collected during prospective national surveillance programs. The surveillance of the NRCMA was approved by the Institut Pasteur Institutional Review Board 1 (approval no. 2009–34/IRB) and the Commission National de l’Informatique et des Libertés, according to French regulations. The YEASTS program collected information and strains corresponding to fungemia episodes in hospitals near Paris during 2002–2022 (*1*). The Réseau de Surveillance des Infections Fongiques (RESSIF) program centralized data on IFD episodes, without selection bias, from 36 hospitals throughout France, diagnosed during 2012 and 2022 (*32*).

We collected demographic and clinical data by using electronic case report form designed on the VOOZANOO platform (http://www2.voozanoo.net). We conducted statistical analyses by using Stata software version 17 (StataCorp, LLC, https://www.stata.com). We expressed categorical variables as percentages and continuous variables as medians +SD. We evaluated differences between the groups by using χ^2^ or Fisher exact tests and considered p values <0.05 statistically significant. We conducted Shapiro-Wilk tests to determine the distribution of MIC values for genotypes 1, 3, and 4 of *T. asahii* and then performed a Kruskal Wallis or analysis of variance test.

### Isolates

As part of this prospective surveillance program, clinical isolates were sent to the NRCMA for complementary investigations, including species identification and in vitro antifungal susceptibility testing by the EUCAST method. Depending on the period, methods for identification were different (ID32C carbon assimilation profiles or matrix-assisted laser desorption/ionization time-of-flight mass spectrometry profiles associated with internal transcribed spacer [ITS] or intergenic spacer [IGS] region sequencing), but when it was possible ITS+IGS regions were sequenced prospectively or retrospectively. For the isolates (n = 3) not sent to the active surveillances programs YEASTS or RESSIF (*32*,*33*) and from before 2008, retrospective identification was not possible.

We amplified ITS regions by PCR by using panfungal primers V9D (5′-TTAAGTCCCTGCCCTTTGTA-3′) (*34*) and LS266 (5′-GCATTCCCAAACAACTCGACTC-3′) (*35*) and the IGS region by using 26SF and 5SR, as previously reported (*14*). We edited sequences by using Geneious Prime software (https://www.geneious.com). We trimmed ITS region sequences with sequences of primers ITS1 (5′-TCCGTAGGTGAACCTGCGG-3′) and ITS4 (5′-GCATATCAATAAGCGGAGGA-3′) and IGS1 region sequences by using sequences SCTTTGSACT and ACYGCATCC, adapted from previous reports (*14*).

For species identification, we compared concatenated sequences of ITS and IGS regions of the clinical isolates with concatenated sequences of type strains. When sequences of isolates had a percentage of similarity with sequence of type strain >98%, we considered isolates as belonging to the same species. When the percentage was 90%–98%, we suggested that isolates could belong to a putative undescribed species, and we named them with a *cf.* in front of the species name. For those isolates, we deposited sequences into GenBank (accession nos. PV575975–8).

We conducted multiple alignments of 106 ITS+IGS concatenated sequences of 1,556 bp (95 clinical isolates and 11 type strains) by using multiple sequence comparison by log-expectation alignment and constructed a neighbor-joining tree by using a Hasegawa-Kishino-Yano model with a bootstrap analysis of 1,000 replicates. We used a newick tree to design a cladogram with Itol software version 1.9 (https://itol.embl.de). We used IGS1 sequences for *T. asahii* isolates to determine genotype.

### Antimicrobial Susceptibility Profile

We determined MICs for fluconazole, voriconazole, posaconazole, and flucytosine for all isolates according to the EUCAST broth microdilution standardized method (https://www.eucast.org/fileadmin/src/media/PDFs/EUCAST_files/AFST/Files/EUCAST_E.Def_7.4_Yeast_definitive_revised_2023.pdf). We also determined caspofungin, micafungin, and amphotericin B MICs with a modified version of the protocol, which uses AM3 medium instead of RPMI medium as described previously (*36*).

We calculated values of MIC inhibiting at least 50% (MIC_50_) or 90% (MIC_90_) of the isolates among 1 species. Of note, neither EUCAST nor Clinical and Laboratory Standards Institute currently publish breakpoints for any Trichosporonacea species. Recently, EUCAST proposed interpretation of amphotericin B and anidulafungin MICs for *Trichosporon* spp.; but because we did not use standard conditions to determine MICs for amphotericin B, we cannot take those values into account. However, epidemiologic cutoffs have been proposed for fluconazole for *T. asahii* and for fluconazole and voriconazole for *C. dermatis* (https://www.eucast.org/fileadmin/src/media/PDFs/EUCAST_files/AFST/Files/EUCAST_guidance_for_Rare_yeast_with_no_breakpoints_final_clean_19-06-2024.pdf).

### Phenotypic Observation

We examined the macroscopic appearance of 10 isolates belonging to species of the ovoides clade, containing the main *Trichosporon* species involved in IFD. Those isolates were CBS 4828 type of *T. faecale*, CNRMA15.795 *T. cf. faecale*, CBS 5585 neotype of *T. inkin*, CNRMA20.443 *T. austroamericanum*, CBS 2482 type of *T. coremiiforme*, CNRMA19.523 *T. cf. coremiiforme*, CBS 7556 neotype of *T. ovoides*, CBS 9051 type of *T. lactis*, CBS 9052 type of *T. caseorum*, and CBS 2479 type of *T. asahii.*

We prepared a suspension concentrated at 2.10^5^ cells/mL on the basis of a fresh culture of isolates in sterile water. We determined concentration by using luna cell counting slides (Logos Biosystems Inc., https://logosbio.com). Then, we deposited 2 µL of the solution on Sabouraud agar plates and incubated at 20°C. We measured the size of the colony after 4, 5, 6, 7, and 10 days and noted color and appearance.

## Results

### Episode Characteristics

During 2002–2022, a total of 112 cases of IFD caused by Trichosporonaceae, mainly bloodstream infection (77.7%), concerning 112 patients, were reported to the NRCMA from 41 hospitals in France (37 metropolitan and 4 overseas) (Table 1). Most patients were male (66.1% vs. 33.9% female); median age was 43.77 years, and a high percentage were extreme ages (18.8% children <15 years of age and 17.9% adults >65 years of age). The main underlying conditions were hematologic malignancies (39.3%), recent surgery (<30 days from diagnosis; 28.6%), and solid organ transplantation (11.6%). Twelve patients also had diabetes mellitus, 7 had traumas (generally serious accidents involving contact with plants, such as being crushed by a tree, or mower or rototiller accidents resulting in multiple fractures or amputations), and 23 were neutropenic. Risk factors associated were stay in ICU (45.9%), presence of catheter (56.3%), immunosuppressive drugs (37.5%), or administration of steroids (22.3%). At least 33.9% of patients received antimicrobial drugs before IFD diagnosis, mainly echinocandins (24.1%). Most patients received antimicrobial drugs after diagnosis (90.2%), mainly with voriconazole (57.1%). Mortality by day 30 after diagnosis was 38.4%.

**Table 1:** Demographic and clinical characteristics of patients in study of clinical manifestations of emerging *Trichosporon* species infections, France, 2002–2022*

Characteristics	Value
Sex	
M	74 (66.1)
F	38 (33.9
Age, years, mean ±SD (range)	43.77 ±24.39 (1–90)
<15 y	21 (18.8)
>65 y	20 (17.7)
Clinical information	
Bloodstream infection	87 (77.7)
Bone infection	12 (10.7)
Stay in intensive care unit, n = 98	45 (45.9)
Underlying conditions	
Hematologic malignancy	44 (39.3)
Acute leukemia	25
Lymphoma	13
Recent surgery	32 (28.6)
Solid organ transplantation	14 (11.6)
Kidney	5
Heart	4
Liver	4
Lung	1
Diabetes	12 (10.7)
Risk factors	
Neutropenia	23 (20.5)
Presence of catheter	63 (56.3)
Immunosuppressive treatment	42 (37.5)
Steroids	25 (22.3)
Antifungal exposure	38 (33.9)
Echinocandins	27 (24.1)
Antifungal treatment	101 (90.2)
Containing voriconazole	64 (57.1)
Containing amphotericin B	24 (21.4)
Outcome, n = 99	
Death within 30 days of diagnosis	38 (38.4)

*Values are no. (%) except as indicated.

Of note, among the 112 episodes, 14 corresponded to mixed infections. Those patients were simultaneously infected with 1 or 2 additional fungal species, mainly ascomycetous yeast (n = 9) at the same infection site (Appendix Table). Those episodes were caused by 6 different species of Trichosporonaceae, although *T. asahii* was involved in most cases (9/14).

### Species Diversity

**Figure 1:**
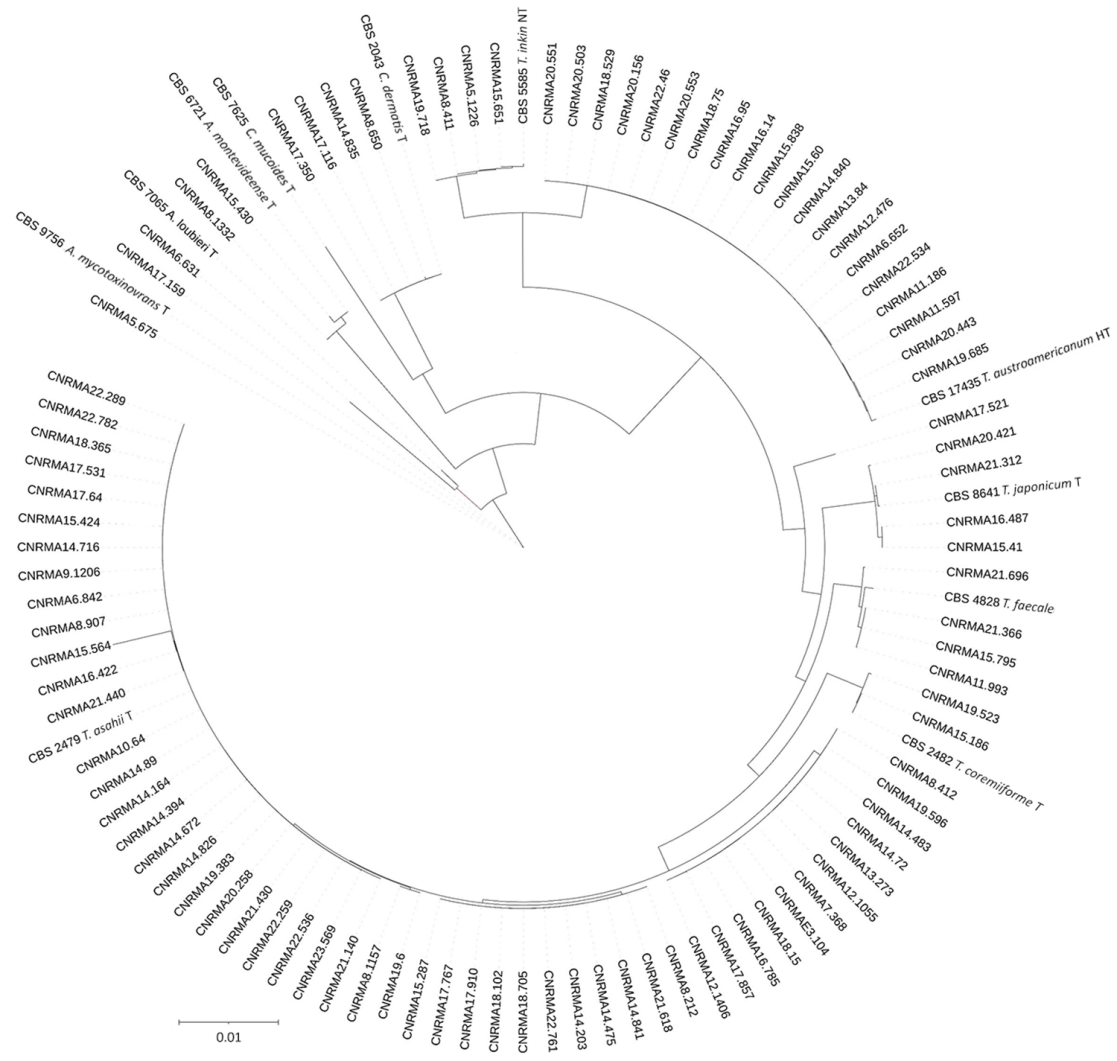
Cladogram of 94 clinical isolates and 11 reference strains from study of clinical manifestations of emerging *Trichosporon* species infections, France, 2002–2022. Tree was designed using Itol software version 1.9 (https://itol.embl.de) on the basis of the newick tree obtained from the Hasegawa-Kishino-Yano model analysis after multiple alignment of concatenation of trimmed sequences of internal transcribed spacer and intergenic spacer regions (Geneious Prime, https://www.geneious.com). GenBank accession numbers or type strain identification numbers are provided for each isolate. Scale bar indicates substitutions per site.

Among the 112 episodes studied, 101 isolates from 101 episodes were sent to the NRCMA. On the basis of the ITS+IGS sequencing, 94 isolates belonged to 9 already described species, distributed across 3 genera (*Trichosporon* [n = 5], *Apiotrichum* [n = 2], *Cutaneotrichosporon* [n = 2]). Seven isolates corresponded to 3 putative unknown species: 4 closely related to *T. faecale* (GenBank accession nos. PV575976 and PV575977), 2 to *T. coremiiforme* (GenBank accession no. PV575975) and 1 to *A. loubierii* (GenBank accession no, PV575978) (Figure 1).

**Figure 2:**
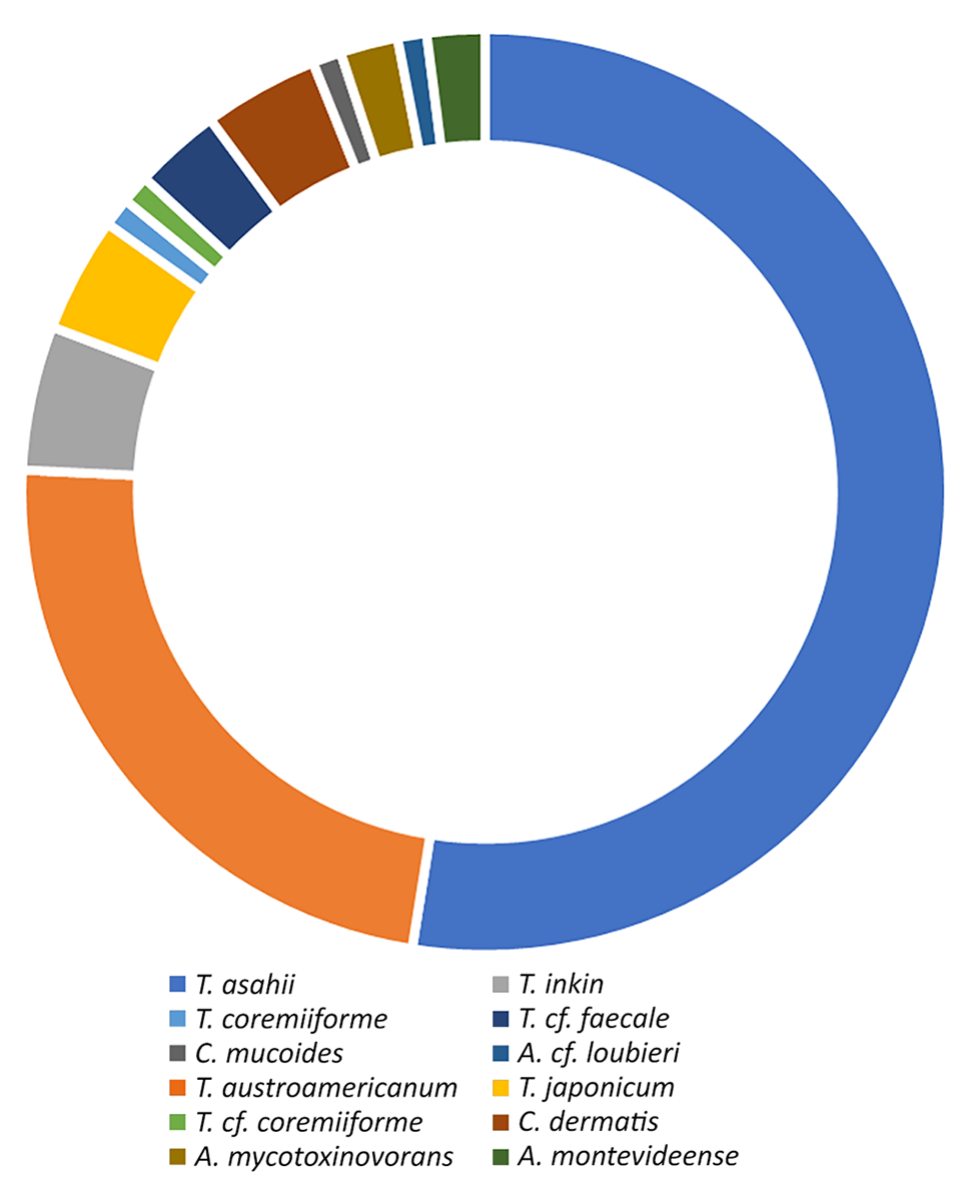
Proportions of species involved in invasive trichosporonosis cases from study of clinical manifestations of emerging *Trichosporon* species infections, France, 2002–2022. The *cf.* designation indicates putative undescribed species.

Most isolates belonged to the *Trichosporon* genus (90.1%). Two species represented most cases of IFD caused by *Trichosporonaceae* in France: *T. asahii* (51.5%) and the species recently described as *T. austroamericanum* (22.8%), which is closely related to *T. inkin* (Figure 2). Among the 58 cases of *T. asahii*, 51 isolates were received at the NRCMA. On the basis of IGS sequencing, 6 genotypes were identified, mainly genotype 1 (n = 25) and genotypes 3 (n = 11) and 4 (n = 11), followed by genotype 7 (n = 2), genotype 5 (n = 1), and 1 isolate with an undefined allele.

**Figure 3:**
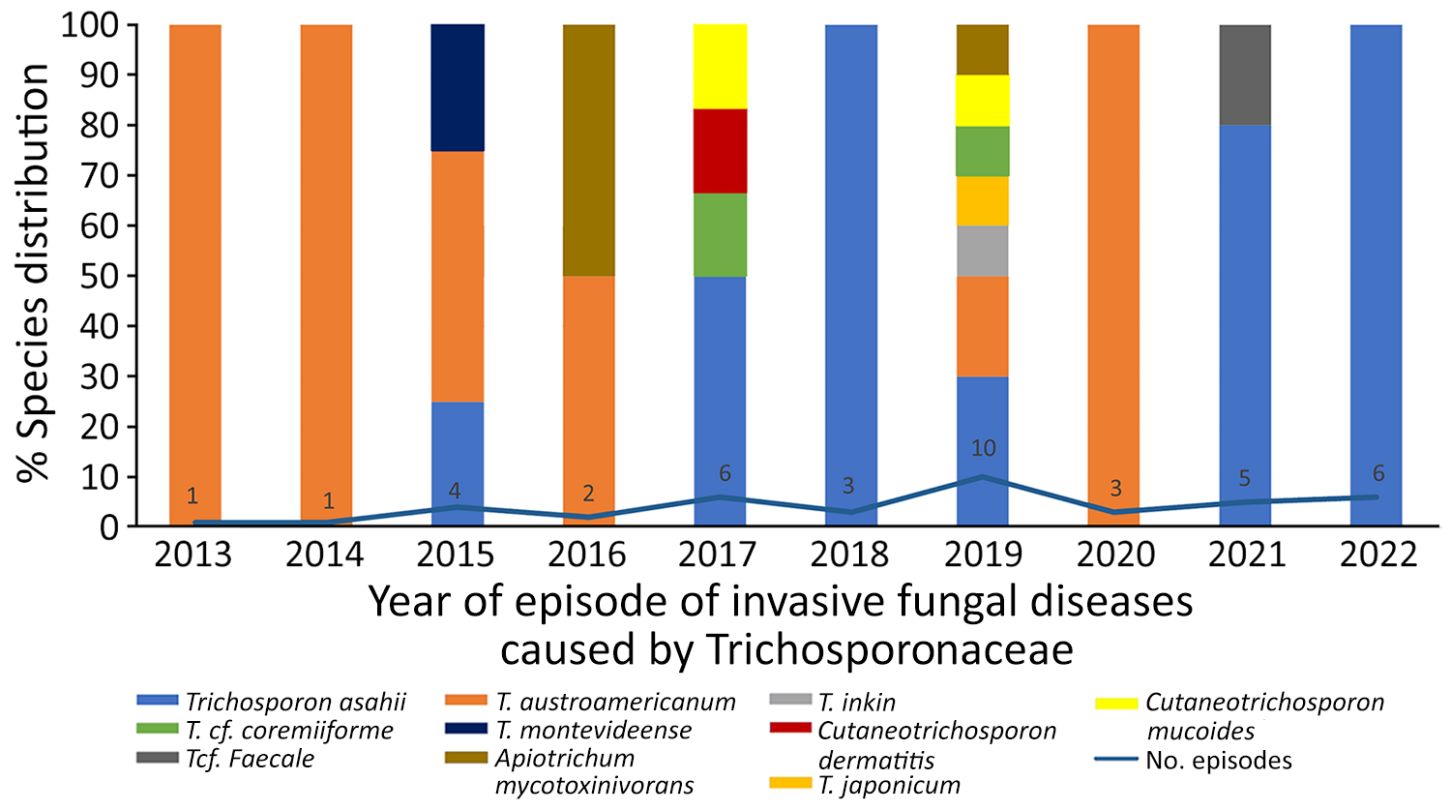
Species distribution over time for RESSIF survey used in a study of the clinical manifestations of emerging *Trichosporon* species infections, France, 2002–2022. Number of episodes diagnosed in the centers participating in the survey are reported for each year. The *cf.* designation indicates putative undescribed species.

Episodes of IFD caused by Trichosporonaceae were reported under several national surveillance programs. If we compare the distribution of species and the number of episodes by year over the same period for the RESSIF program and the YEASTS program, the proportion of *T. asahii* is higher among the YEASTS program than among the RESSIF survey but the opposite for *T. austroamericanum.* A trend for an increased number of episodes over time in the RESSIF survey was seen (Figure 3).

### In Vitro Antimicrobial Drug Susceptibility

**Figure 4:**
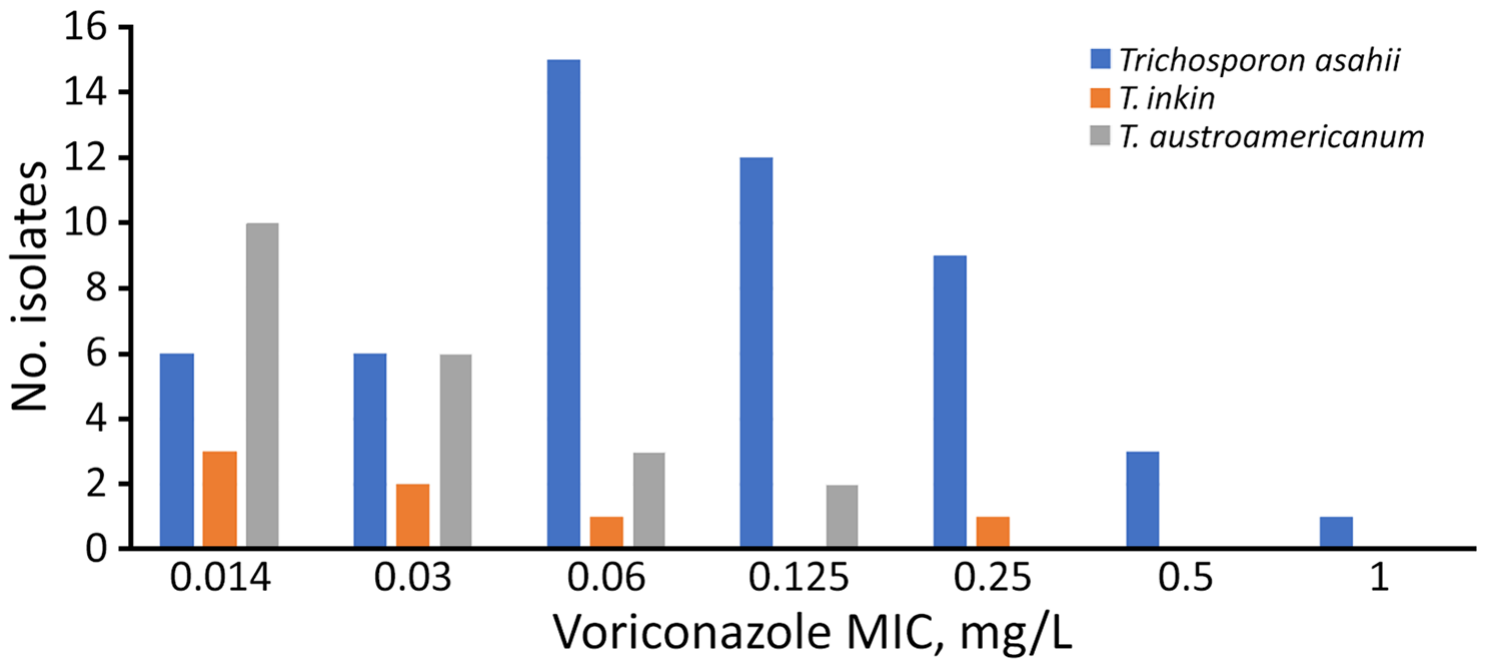
Voriconazole MIC distribution for 3 species of *Trichosporon* from study of clinical manifestations of emerging *Trichosporon* species infections, France, 2002–2022. MICs determined by using the EUCAST method.

Voriconazole appears to be the most active antifungal agent in vitro (Table 2), with the lowest MIC_50_ or MIC_90_ values and ranges of MICs for all species, except for *A. mycotoxinivorans*, for which both isolates have lower values for posaconazole than voriconazole. Conversely, all isolates of all species have high MICs to flucytosine (>4 mg/L and all MIC_50_ determined ≥32 mg/L) and to echinocandins (data not shown). *Trichosporon asahii*, *A. loubieri*, *A. mycotoxinivorans*, and isolates closely related to *T. faecale* have reduced susceptibility to amphotericin B compared with other Trichosporonaceae species. More precisely, *T. asahii* isolates belonging to genotype 4 tend toward lower MIC_50_ or MIC_90_ values for azoles and amphotericin B than isolates of genotypes 1 and 3 (genotype 1 displayed the highest MIC_50_ or MIC_90_ for all antimicrobial drugs), but the differences were statistically significant only for amphotericin B (p = 0.0073). EUCAST recently defined epidemiologic cutoff values for fluconazole (wild-type <16 mg/L) for *T. asahii* and for fluconazole (wild-type <16 mg/L) and voriconazole (wild-type <0.125 mg/L) for *C. dermatis*. Taking those values into account, we can see only 4 isolates of genotype 1 can be considered resistant to fluconazole. We found no resistant isolates among the other genotypes or for *C. dermatis* strains. Furthermore, *T. asahii* have higher MICs for azoles and amphotericin B than *T. austroamericanum* and *T. inkin* with a difference in voriconazole MIC distribution (Figure 4).

**Table 2:** Antimicrobial susceptibility profiles of 101 clinical isolates determined by using the EUCAST method, according to species and genotypes recovered, in study of clinical manifestations of emerging *Trichosporon* species infections, France, 2002–2022*

Species and genotype (no. isolates)	MIC_50_/MIC_90_, mg/L (range of MICs)				
	AMB	5FC	FLUCO	VORI	POSA
*Trichosporon asahii* (52)	2/>4 (0.25–>4)	64/>64 (4–>64)	4/16 (0.25–64)	0.06/0.25 (<0.015–1)	0.25/0.5 (<0.015–1)
Genotype 1 (25)	2/>4 (0.25–>4)	64/>64 (4–>64)	4/32 (0.25–64)	0.125/0.25 (<0.015–1)	0.25/0.5 (<0.015–1)
Genotype 3 (12)	1/2 (0.5–4)	32/>64 (8–>64)	1 /4 (0.5–8)	0.06/0.25 (0.03–0.5)	0.25/0.5 (0.06–0.5)
Genotype 4 (11)	1/1 (0.25–2)	32/>64 (8–>64)	2/4 (0.25–16)	0.06/0.125 (<0.015–0.25)	0.06/0.25 (<0.015–0.5)
Genotype 7 (2)	−/− (2)	−/− (>64)	−/− (2–16)	−/− (0.06–0.5)	−/− (0.03–0.5)
Genotype 5 (1)	−/− (4)	−/− (32)	−/− (2)	−/− (0.03)	−/− (0.25)
Genotype 13† (1)	−/− (>4)	−/− (>64)	−/− (8)	−/− (0.125)	−/− (1)
*T. austroamericanum* (21)	0.25/1 (0.06–2)	>64/>64 (64–>64)	0.5/4 (0.25–4)	0.03/0.06 (<0.015–0.125)	0.06/0.25 (<0.015–0.25)
*T. inkin* (7)	0.5/− (0.125–>4)	>64/− (16–>64)	2/- (1–16)	0.03/− (<0.015–0.25)	0.125/− (<0.015–0.5)
*T. cf. coremiiforme* (2)	−/− (0.5–2)	−/− (8–32)	−/− (0.5–1)	−/− (0.03–0.125)	−/− (0.06–0.125)
*T. coremiiforme* (1)	−/− (0.5)	−/− (16)	−/− (2)	−/− (0.03)	−/− (0.125)
*T. cf. faecale* (4)	−/− (1–>4)	−/− (16–>64)	−/− (1–16)	−/− (0.03–0.25)	−/− (0.03–0.5)
*T. japonicum* (4)	−/− (0.25–4)	−/− (8–>64)	−/− (0.5–4)	−/− (<0.015–0.125)	−/− (0.03–0.125)
*Cutaneotrichosporon dermatis* (4)	−/− (0.06–0.25)	−/− (>64)	−/− (2–16)	−/− (0.03–0.125)	−/− (<0.015–0.125)
*C. mucoides* (1)	−/− (0.06)	−/− (8)	−/− (2)	−/− (0.125)	−/− (0.5)
*Apiotrichum loubieri* (1)	−/− (1)	−/− (8)	−/− (2)	−/− (0.03)	−/− (0.06)
*A. montevideense* (2)	−/− (0.06–0.25)	−/− (>64)	−/− (1)	−/− (0.03–0.06)	−/− (0.03–0.125)
*A. mycotoxinivorans* (2)	−/− (2)	−/− (16–32)	−/− (2–8)	−/− (0.125–1)	−/− (<0.015–0.25)

*AMB, amphotericin B; *cf*., putative undescribed species; FLUCO, fluconazole; POSA, posaconazole; VORI, voriconazole; 5FC, flucytosine. †Genotype 13 corresponds to the genotype described previously (GenBank accession no. MN936175)

### Growth Rate and Morphologic Aspect

**Figure 5:**
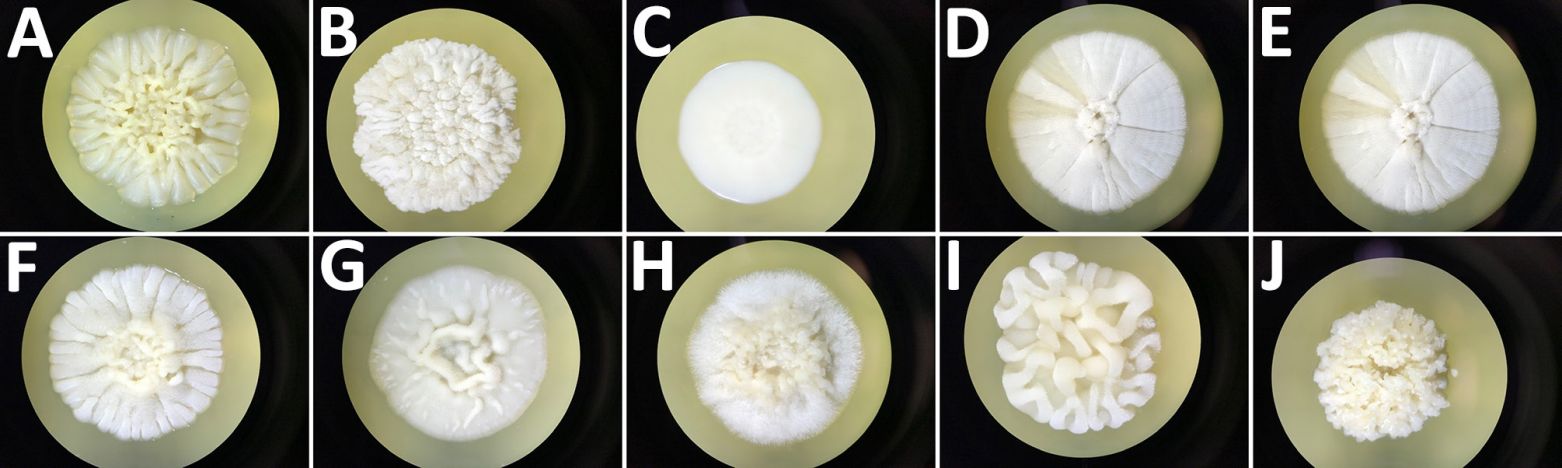
Macroscopic aspect of colonies grown on Sabouraud agar plates incubated 10 days at 20°C for 10 isolates recovered from study of clinical manifestations of emerging *Trichosporon* species infections, France, 2002–2022. A) *T. asahii* type strain isolate CBS 2479. B) *T. austroamericanum* isolate CNRMA20.443. C) *T. inkin* neotype strain isolate CBS 5585. D) *T. coremiiforme* type strain isolate CBS 2482*.* E) *T. cf. coremiiforme* isolate CNRMA19.523. F) *T. faecale* type strain isolate CBS 4828. G) *T. cf. coremiiforme* isolate CNRMA15.795*.* H) *T. ovoides* neotype strain isolate CBS 7556*.* I) *T. caseorum* type strain isolate CBS 9052. J) *T. lactis* isolate CBS 9051*.* *T. coremiiforme* and isolates corresponding to the potential *T. cf. coremiiforme* species (E, G) had the highest growth rate, whereas *T. inkin* and *T. austroamericanum* had reduced but similar growth rate. The *cf.* designation indicates putative undescribed species.

On the basis of the growth rate measured for 10 isolates belonging to different species of *Trichosporon* (Appendix Figure), we observed that *T. coremiiforme* and isolates corresponding to the potential *T. cf. coremiiforme* (isolate name CNRMA19.523) species had the highest growth rate, whereas *T. inkin* and *T. austroamericanum* had reduced but similar growth rate. All isolates grew as white colonies on Sabouraud agar plate and were generally creamy, although some species appeared smooth or flat to domed and wrinkled (Figure 5).

### *T. asahii* versus *T. austroamericanum*

Episodes involving only 1 species (excluding mixed infections) and those with isolates sent to NRCMA were included in this comparison, which corresponds to 44 episodes of *T. asahii* infection and 22 of *T. austroamericanum*. A higher proportion of women and children among the patients infected with *T. asahii* were observed, whereas the proportion of men was much higher among patients infected with *T. austroamericanum.* Furthermore, infections because of *T. austroamericanum* were more frequently associated with recent surgery (p = 0.03) or solid organ transplantation (p = 0.008) (Table 3).

**Table 3:** Comparison of demographic and clinical characteristics of the patients with single trichosporonosis episodes caused by *Trichosporon asahii* versus *T. austroamericanum* from study of clinical manifestations of emerging *Trichosporon* species infections, France, 2002–2022*

Characteristics	*T. asahii,* n = 44	*T. austroamericanum*, n = 22	p value
Sex			
M	23 (52.2)	19 (86.4)	0.007
F	21 (47.8)	3 (13.6)	
Mean age, y, ±SD (range)	43 ±24.5 (1–83)	50 ±19.7 (8–83)	0.159
<15 y	13 (29.5)	1 (4.5)	0.024
>65 y	6 (13.6)	5 (22.7)	0.35
Site of infection			
Bloodstream infection	37 (84.1)	15 (68.2)	0.038
Bone	3 (6.8)	4 (18.2)	
Skin	3 (6.8)	1 (4.5)	
Intensive care unit admission	15 (34.1)	8 (36.4)	0.903
Hematologic malignancy	22 (50)	6 (27.3)	0.082
Acute leukemia	12 (27.3)	3 (13.6)	
Lymphoma	8 (18.2)	1 (45)	
Recent surgery	6 (13.6)	8 (36.4)	0.03
Orthopedic	2 (4.5)	2 (9.1)	
Cardiac	0	2 (9.1)	
Kidney-urinary tract	0	2 (9.1)	
Solid organ transplantation	3 (6.8)	8 (36.4)	0.008
Liver	2 (4.5)	1 (4.5)	
Kidney	1 (2.3)	3 (13.6)	
Heart	0	3 (13.6)	
Lung	0	1 (4.5)	
Presence of catheter	29 (65.9)	10 (45.5)	0.315
Exposure to antimicrobial drugs	13 (29.5)	10 (45.5)	0.175
Neutropenia	11 (25)	3 (13.6)	0.535
Immunosuppresive drugs	19 (43.2)	11 (50)	0.571
Corticotherapy	12 (27.3)	4 (18.2)	0.665
Death within 30 days of diagnosis	14 (31.8)	5 (22.7)	0.389

*Values are no. (%) except as indicated.

## Discussion

In this report, we describe demographic, clinical, and molecular characteristics of a large collection of 112 cases of IFD caused by Trichosporonaceae fungi diagnosed in France throughout 2 decades during multicentric national prospective surveillance programs. As mentioned in other studies, we observed a high proportion of children among the patients (18.8%) but with a significant difference in proportion depending on the species (*26*). As outlined in this study, most cases of IFD caused by Trichosporonaceae are bloodstream infections (77.7%) and occur in patients with underlying conditions such as hematologic malignancy (39.3%), recent surgery (28.6%), or solid organ transplant (11.6%) (*1*,*2*,*28*). A recent study concluded that, in cases of fungemia caused by *Trichosporon* spp., advanced age, use of mechanical ventilation, and persistent neutropenia were associated with poor prognosis (*4*). Another study demonstrated that exposure to caspofungin is a risk factor associated with fungemia caused by *Trichosporon* spp. (*1*). Similarly, the risk factors that we found frequently were hospitalization in ICU (45.9%), presence of catheter (56.3%), and exposure to antimicrobial drugs (33.9%), primarily echinocandins (27/38).

On the basis of the data from RESSIF, we seem to be observing a trend in France toward an increase in the number of cases of IFD caused by Trichosporonaceae. However, the incidence of those infections is difficult to determine precisely, because some participating centers did not participate exhaustively over the entire study period.

Sequencing of ITS+IGS regions of rDNA enabled us to identify >12 different species of Trichosporonaceae responsible for 112 IFD cases in 41 different hospitals in France during 2002–2022. Of note, all cases reported from overseas territories were because of *T. asahii* only. We suggest that 7 cases were because of putative undescribed species, for which additional molecular characterizations such as whole-genome sequencing analysis are now warranted. As expected, most cases were caused by *T. asahii*, but unexpectedly, the recently described species *T. austroamericanum* ranked second (*12*). The percentage of children was significantly lower among patients infected with this new species (4.5%) than among those infected with *T. asahii* (29.5%; p = 0.024). On the other hand, the percentage of men was much higher (86% vs. 52%; p = 0.007). In addition, *T. asahii* was more frequently involved in bloodstream infections or exposure to an antimicrobial drug, whereas *T. austroamericanum* was more frequently associated with recent surgery (p = 0.03) and solid organ transplant (p = 0.008) (Table 3).

Sequencing of the IGS region for *T. asahii* isolates is described as a useful tool for genotyping this species. At least 13 different genotypes have already been identified, some of which appear to have geographic preferences. Genotypes 1, 3, and 4 seem to be more frequent worldwide (*5*,*15*,*16*,*23*,*37*). Among the 101 isolates received at the NRCMA during the study period we report, the 51 isolates of *T. asahii* belonged to 6 different genotypes, and we confirmed the major genotype was 1 (49%), followed by genotypes 3 (21%) and 4 (21%). Of note, genotype 3 is frequently reported in the United States, in Thailand, and in the houses of summer-type hypersensitivity pneumonitis patients in Japan (*14*,*17*). Of interest, among the 11 patients infected with genotype 3 in this study, at least 6 were recovered from patients born or diagnosed in the Americas. Surprisingly, according to the genotype and excluding cases of mixed infections, 13 of 21 patients infected by genotype 1 died within 30 days of diagnosis: 5 of 11 for genotype 3 and only 1 of 8 for genotype 4. Those results could suggest some epidemiologic characteristic related to genotype or some difference in terms of virulence.

We confirmed that no matter the species or genotypes, voriconazole is the most effective antimicrobial drug in vitro against isolates belonging to the Trichosporonaceae family (*17*,*20*). Because of the small number of isolates for 10 species, we can only give the MIC values obtained for informational purposes. However, there are some differences worth noting: *T*. *asahii*, *A. loubieri*, *A. mycotoxinivorans*, and the isolates closely related to *T. faecale* have a lower susceptibility to amphotericin B compared with other species. Furthermore, *T. asahii* has higher MICs for azoles and amphotericin B than do *T. austroamericanum* and *T. inkin*. Some differences are noticed between genotypes; genotype 1 has the highest MIC for all antimicrobial drugs tested, whereas genotype 4 has lower MICs to azoles and amphotericin B than isolates of genotypes 1 and 3. Some studies reported a lower susceptibility for certain genotypes (*17*,*37*,*38*) and one study found that genotype 7 isolates have the highest MIC90 values for azoles, suggesting those isolates contributed to the increasing rates of voriconazole non-wild–type isolates observed in the past 10 years (*37*). In this study, the proportion of genotype 7 was too small to calculate MIC_90_s. Nevertheless, determining natural diversity would be useful to explain differences in susceptibility or even virulence, possibly linked to environmental pressures such as repeated exposure to azoles, because that mechanism is known in other pathogenic fungi (*39*–*41*).

We confirmed that *T. asahii*, especially genotype 1, is the major species of the Trichosporonaceae family involved in human IFD. We also confirmed that the pediatric population is at a higher risk and that the most frequent underlying conditions associated with infection are hematologic malignancies, recent surgery, and solid organ transplantation. We identified 3 main genotypes recovered among the patients diagnosed in French hospitals (genotypes 1, 3, and 4). Of note, we observed that genotype 4 seems to be more frequently associated with trauma and having lower MICs values for amphotericin B and azoles, compared with other genotypes. Species and genotype identification of environmental isolates will be necessary to increase the knowledge about natural reservoirs, diversity, and virulence.

We observed that the recently described *T. austroamericanum*, closely related to *T. inkin* and frequently misidentified by using carbon profile assimilation pattern, 26S, ITS sequencing, or matrix-assisted laser desorption/ionization time-of-flight mass spectrometry, was the second most common Trichosporonaceae family involved in human IFD. When compared with the clinical data of patients infected with *T. asahii,* we found very different species characteristics. In contrast, *T. inkin*, *T. faecale*, and *C. dermatis*, which are frequently identified as responsible for non–*T. asahii* IFD, were rarely involved in our survey (*4*,*17*,*21*).

In conclusion, this study confirms that the correct identification of Trichosporonaceae species and genotypes is not only of epidemiologic interest but also critical for patient management. Certain clinical and microbiological characteristics, such as in vitro susceptibility to antimicrobial agents, can vary according to species. Clinicians must have the correct organism identification to treat patients correctly. 

Appendix Additional information about clinical manifestations of emerging *Trichosporon* species infections, France.
