# Supplementary material for: Clinical Manifestations of Emerging Trichosporon spp. Infections, France
Source: Emerg Infect Dis. 2026 Jan;32(1):93–102. doi: 10.3201/eid3201.250504 (PMC12870045; doi:10.3201/eid3201.250504)
Supplement: Appendix — Additional information about clinical manifestations of emerging Trichosporon species infections, France. [file 25-0504-Techapp-s1.pdf]

Article DOI: https://doi.org/10.3201/eid3201.250504

EID cannot ensure accessibility for supplementary materials supplied by authors. Readers who have difficulty accessing supplementary content should contact the authors for assistance.

Clinical Manifestations of Emerging *Trichosporon* Species Infections, France

Appendix

Appendix Table. Demographic and clinical characteristics of the 14 mixed infections

Isolation year	Age	Sex	Underlying disease	Risk factor	Isolation site	Species (genotype)	Other species	Treatment	Death before 3 months
2009	1	F	NA	biliary cyst drains	blood	*T. asahii* (1)	*Fusarium oxysorum (bile)*	voriconazole	yes
2013	52	M	cirrhosis	NA	blood	*T. asahii* (3)	*Candida tropicalis*	amphotericinB	yes
2013	19	M	NA	wound infection	blood	*T. asahii* (NA)*	*Candida parapsilosis*	caspofungin	no
2014	44	M	NA	Bacterial infection	CSF	*T. asahii* (1)	*Candida albicans*	NA*	yes
2015	51	F	lymphoma	NA	blood	*T. inkin*	*Candida tropicalis + Pichia kudriavzevii*	caspofungin	yes
2015	72	M	lymphoma	NA	blood	*T. asahii* (1)	*Clavispora lusitaniae*	caspofungin	yes
2017	47	F	NA	Extensive burn	blood	*T. asahii* (4)	*Candida orthopsilosis*	fluconazole+caspofungin	NA
2019	55	M	NA	Plant trauma	thigh soft tissue	*T. japonicum*	*Trichoderma longibrachiatum + Clavispora lusitaniae*	voriconazole	no
2019	67	M	Acute leukemia	NA	blood	*T. asahii* (3)	*Candida parapsilosis*	amphotericinB	yes
2019	64	M	lymphoma	NA	blood	*T. asahii* (1)	*Pichia kudriavzevii*	voriconazole	yes
2019	39	M	NA	Grass shredder trauma	biopsy of foot soft tissue	*C. mucoides*	*Alternaria sp*	voriconazole	no
2019	89	M	NA	tiller accident	biopsy of leg soft tissue	*A. mycotoxinivorans*	*Rhizopus sp*	amphotericinB	no
2020	60	M	kidney transplant	NA	blood	*T. austroamericanum*	*Candida parapsilosis + Aspergillus fumigatus (asp trachéale)*	voriconazole	yes
2022	66	M	Acute leukemia	NA	blood	*T. asahii* (1)	*Kluyveromyces marxianus*	caspofungin	yes

*NA not available

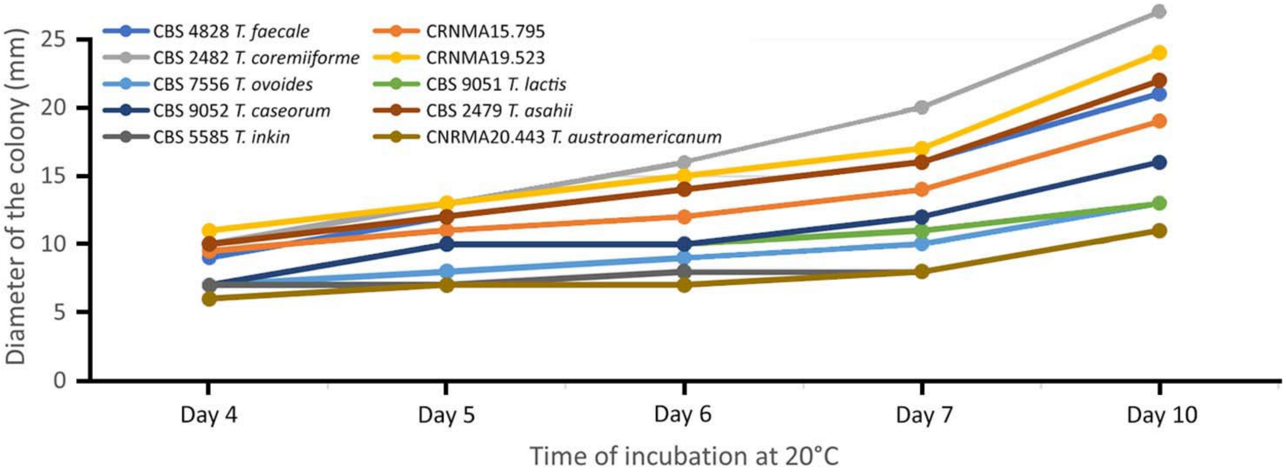

Appendix Figure. Size of colonies on Sabouraud agar plate incubated at 20°C
